# Critical Role for Molecular Iron in Coxiella burnetii Replication and Viability

**DOI:** 10.1128/mSphere.00458-20

**Published:** 2020-07-22

**Authors:** Savannah E. Sanchez, Anders Omsland

**Affiliations:** a Paul G. Allen School for Global Animal Health, Washington State University, Pullman, Washington, USA; b School of Molecular Biosciences, College of Veterinary Medicine, Washington State University, Pullman, Washington, USA; University of Kentucky

**Keywords:** *Coxiella burnetii*, axenic, virulence, iron, bacteriology, physiology

## Abstract

Coxiella burnetii, the causative agent of Query (Q) fever in humans, is a highly infectious obligate intracellular bacterium. Following uptake into a host cell, C. burnetii replicates within a phagolysosome-derived compartment referred to as the *Coxiella*-containing vacuole (CCV). During infection, C. burnetii exhibits tropism for tissues related to iron storage and recycling (e.g., the liver and splenic red pulp), suggesting that pathogen physiology is linked to host iron metabolism. Iron has been described to have a limited role in C. burnetii virulence regulation, despite evidence that C. burnetii*-*infected host cells increase expression of transferrin receptors, thereby suggesting that active iron acquisition by the bacterium occurs upon infection. Through the use of host cell-free culture, C. burnetii was separated from the host cell in order to directly assess the role of different forms of iron in C. burnetii replication and viability, and therefore virulence. Results indicate that C. burnetii tolerates molecular iron over a broad concentration range (i.e., ∼0.001 to 1 mM) and undergoes gross loss of viability upon iron starvation. C. burnetii protein synthesis and energy metabolism, however, occur nearly uninhibited under iron concentrations not permissive to replication. Despite the apparent absence of genes related to acquisition of host-associated iron-containing proteins, C. burnetii replication is supported by hemoglobin, transferrin, and ferritin, likely due to release of iron from such proteins under acidic conditions. Moreover, chelation of host iron pools inhibited pathogen replication during infection of cultured cells.

**IMPORTANCE** Host organisms restrict the availability of iron to invading pathogens in order to reduce pathogen replication. To counteract the host’s response to infection, bacteria can rely on redundant mechanisms to obtain biologically diverse forms of iron during infection. C. burnetii appears specifically dependent on molecular iron for replication and viability and exhibits a response to iron akin to bacteria that colonize iron-rich environments. Physiological adaptation of C. burnetii to the unique acidic and degradative environment of the CCV is consistent with access of this pathogen to molecular iron.

## Abstract (precis)

Host organisms restrict the availability of iron to invading pathogens in order to reduce pathogen replication. To counteract the host’s response to infection, bacteria can rely on redundant mechanisms to obtain biologically diverse forms of iron during infection. C. burnetii appears specifically dependent on molecular iron for replication and viability and exhibits a response to iron akin to bacteria that colonize iron-rich environments. Physiological adaptation of C. burnetii to the unique acidic and degradative environment of the CCV is consistent with access of this pathogen to molecular iron.

## INTRODUCTION

Iron is an essential micronutrient often used as a cofactor in enzymes associated with diverse cellular processes, including respiration and DNA biosynthesis (1, 2). Although essential, iron is highly reactive and can lead to generation of oxygen radicals—for example via Fenton chemistry—that are damaging to nucleic acids, proteins, and lipids (3–6). To prevent iron-related toxicity, the concentration of (free) iron is therefore tightly regulated in biological systems. In eukaryotes, iron is largely bound to specific proteins (e.g., ferritin, transferrin, and heme[oglobin]) or complexed by intracellular molecules (e.g., citrate, peptides, ATP and AMP, and pyrophosphates), thereby reducing the amount of free iron (7).

Iron availability in mammals is so tightly regulated that many bacterial pathogens have evolved to maintain redundant mechanisms to adeptly scavenge for this vital micronutrient. These mechanisms include bacterial high-affinity iron transporters and siderophores, iron acquisition receptors of host-associated iron-containing proteins (e.g., lactoferrin via lactoferrin-binding proteins LbpA and LbpB), and direct uptake of bound forms of host iron (e.g., heme/hemoglobin and ferric citrate) (8–10). Moreover, many pathogenic bacteria have evolved to integrate regulation of virulence with iron availability. For instance, the transcription factor ferric-uptake regulator (Fur) mediates overall iron metabolism based on iron availability, including activation of genes involved in redox stress resistance, repair of redox damage (8), and expression of virulence genes (11). Replication is a key aspect of virulence, and access to iron is an absolute requirement for most bacteria (8, 10). Because iron is largely found complexed within biological systems and the mechanism for iron acquisition differs between pathogens, the link between iron utilization and virulence is specific to the pathogen and its niche within the infected host.

Coxiella burnetii, the causative agent of Query (Q) fever in humans and coxiellosis in livestock (e.g., cattle, sheep, and goats), is a highly infectious zoonotic obligate intracellular bacterium (12, 13). Q fever largely presents as a self-limiting and acute febrile disease; however, as many as 5% of cases can progress to chronic Q fever (14), which can require 2 or more years of antibiotic therapy to remedy (15). During infection, C. burnetii replicates exclusively within a host-derived compartment referred to as the *Coxiella*-containing vacuole (CCV) (16, 17). The CCV is a modified phagolysosome that retains key features of this organelle, including a mildly acidic pH (i.e., pH 4.5 to 5.5) (18–20) and acid hydrolases that play an important role in degradation and recycling of host cell components (e.g., macromolecules). During animal infection, C. burnetii exhibits tropism for tissues directly related to iron storage and recycling (e.g., the liver and splenic red pulp) (21, 22), suggesting that pathogen physiology is tied to host iron metabolism.

Iron was previously reported to have a limited role in C. burnetii virulence regulation (23), despite evidence that C. burnetii*-*infected host cells increase receptor expression for the circulating iron-containing protein transferrin (24), thereby suggesting that active iron acquisition by the bacterium occurs during infection. While the C. burnetii genome sequence does not encode siderophores or uptake systems for iron-containing proteins and complexed iron, the pathogen’s genome does encode the ferrous iron uptake transporter FeoAB (C. burnetii RSA 493 CBU1766-1767). In Legionella pneumophila, a close phylogenetic relative of C. burnetii, FeoAB functions in ferrous iron (Fe^2+^) transport (25), suggesting that Fe^2+^ is also the natural iron source for C. burnetii (23).

In the present study, we evaluated the requirement and utilization of iron for C. burnetii replication and viability using host cell-free (axenic) culture tools. Specifically, we took advantage of the natural chelation properties of citrate in order to assess pathogen responses to iron. Our results indicate that while C. burnetii tolerates a wide concentration range of iron, the bacterium appears to require unsequestered, molecular iron. This observation remained apparent during C. burnetii infection of host cells, as sequestration of host iron pools via 2,2′-bipyridyl directly inhibits C. burnetii replication, indicating that C. burnetii relies on its host’s labile iron pool (LIP).

## RESULTS

### C. burnetii exhibits a physiologically dynamic response to iron availability.

**FIG 1:**
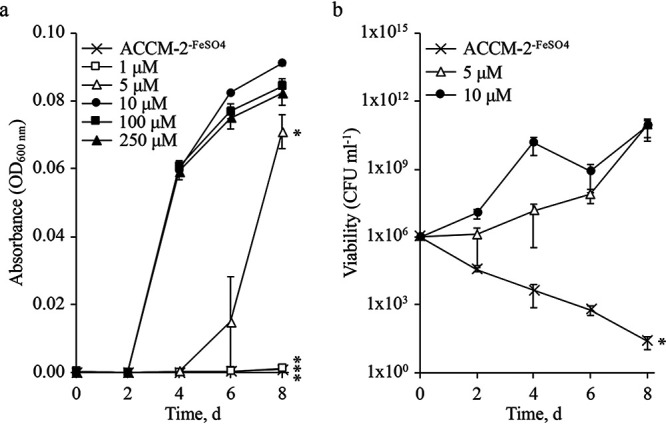
Replication and viability of C. burnetii are dynamically responsive to iron availability. C. burnetii iron utilization was tested in ACCM-2. Media supplemented with various concentrations of FeSO_4_ were monitored every 2 days for 8 days to determine C. burnetii replication by measuring absorbance (a) and viability by CFU enumeration (b). Control conditions for ACCM-2 include FeSO_4_ at a final concentration of 10 μM. Data points reflect the average of 3 independent experiments; error bars indicate SEM. *, *P* < 0.05; ***, *P* < 0.0001. Statistical significance was determined by comparison to control conditions for replication and by comparing starting versus final CFUs for viability (unpaired Student’s *t* test).

Previous analyses on the importance of iron in C. burnetii have been based on the use of C. burnetii-infected host cells (23, 24). To assess the importance of iron in C. burnetii replication, and therefore virulence, we utilized axenic culture tools to separate the bacterium from the host and thus assess the direct effect of iron on the pathogen. While most analyses of bacterial iron acquisition and utilization rely on the use of chemical iron chelators (e.g., 2,2′-bipyridyl or deferoxamine), the citrate-based nutrient medium ACCM-2 (26, 27) used to cultivate C. burnetii allows control of iron availability due to the iron-binding properties of citrate. C. burnetii was cultured in ACCM-2 not supplemented with iron sulfate (FeSO_4_) (ACCM-2^−FeSO4^)—determined to contain ∼1 μM iron by inductively coupled plasma mass spectroscopy (ICP-MS) (see Fig. S1a in the supplemental material)—or supplemented with a specific concentration of FeSO_4_ and incubated for 8 days, with culture development measured every 2 days. Compared to control conditions (i.e., 10 μM FeSO_4_), C. burnetii was unable to replicate when the medium was supplemented with 1 μM FeSO_4_ (Fig. 1a). In contrast, replication of C. burnetii in ACCM-2 supplemented with 5 μM FeSO_4_ was suboptimal and resulted in a 4-day delay in replication and reduced final yields compared to the control (Fig. 1a). In the presence of 100 or 250 μM FeSO_4_, C. burnetii replication exhibited kinetics similar to that observed under control conditions (Fig. 1a).

10.1128/mSphere.00458-20.1 FIG S1 ICP-MS was used to determine the relative concentrations of iron in APCM^−FeSO4^, ACCM-2^−FeSO4^, and (plain) media supplemented with 5 or 10 μM FeSO_4_ (a). C. burnetii was grown axenically in ACCM-2 containing different concentrations of FeSO_4_ or iron chloride (FeCl_3_) (b). Each bar represents the average from 3 independent experiments; error bars indicate SEM. Download FIG S1, PDF file, 0.07 MB. Copyright © 2020 Sanchez and Omsland. 2020 Sanchez and Omsland This content is distributed under the terms of the Creative Commons Attribution 4.0 International license.

To confirm a critical role for iron in FeSO_4_, C. burnetii was incubated in ACCM-2^−FeSO4^ or in medium supplemented with FeSO_4_ or iron chloride (FeCl_3_). Following an 8-day incubation, final yields were determined via quantification of genome equivalents (GE). As expected, FeCl_3_ supported similar C. burnetii yields as FeSO_4_, eliminating a critical role for the SO_4_^2−^ supplied via FeSO_4_ in C. burnetii growth (Fig. S1b). Furthermore, these data suggest that *Coxiella* can tolerate iron at concentrations far exceeding those required for replication (i.e., up to 1 mM) with only a marginal reduction in final yields (Fig. S1b).

Because of the significant difference between C. burnetii growth kinetics under different iron availabilities (Fig. 1a), we sought to determine whether viability was similarly dependent on the concentration of iron. Therefore, CFUs were enumerated every 2 days over an 8-day incubation. In the absence of FeSO_4_, CFUs declined by ∼3 and 5 logs within 4 and 8 days, respectively (Fig. 1b). In contrast, under control conditions, CFUs increased over time (Fig. 1b). In the presence of 5 μM FeSO_4_, C. burnetii CFUs remained stable for the first 2 to 4 days and then increased to reach final CFUs equivalent to control conditions (Fig. 1b).

### Citrate plays a critical role in iron-dependent axenic replication of C. burnetii.

**FIG 2:**
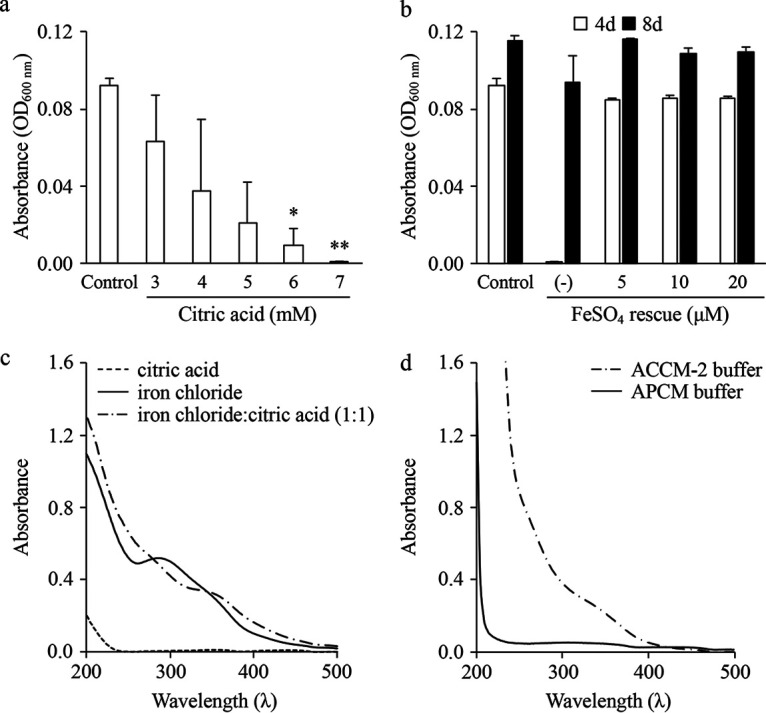
C. burnetii has a reduced ability to utilize iron complexed with citrate. To evaluate the ability of C. burnetii to acquire and utilize iron-citrate, the alternative axenic medium APCM was supplemented with various concentrations of citric acid (a) and C. burnetii growth was determined by measuring absorbance after 4 days. To determine whether the inhibitory effect of citric acid could be rescued by iron supplementation, APCM cultures containing 7 mM citric acid were supplemented with additional FeSO_4_ and C. burnetii final yields were measured via absorbance (b). Each bar represents the average from 2 independent experiments; error bars indicate SEM. *, *P* < 0.05; **, *P* < 0.01 (unpaired Student’s *t* test versus control conditions). To confirm the presence of iron-citrate complexes under the conditions tested, the UV-visible spectra for solutions of citric acid, iron chloride, or a mixture of iron chloride and citric acid (c) were compared to spectra obtained using APCM and ACCM-2 basal buffers (d). With the exception of APCM, plotted spectra represent the average from 5 independent scans. Data from a representative experiment are shown.

The data presented in Fig. 1 suggested C. burnetii requires 5 to 10 μM molecular iron in order to replicate optimally. To test the ability of C. burnetii to replicate in medium containing less than 5 μM molecular iron, a medium free of citrate, acidified phosphate *Coxiella* medium (APCM), was used. The basal buffer for APCM is based in part on the previously described P-25 buffer (28). Dose-response analysis with FeSO_4_ in APCM showed that C. burnetii could replicate with similar growth kinetics regardless of iron supplementation (Fig. S2a). Importantly, C. burnetii cultures reached high turbidities even at 0.5 mM FeSO_4_, as seen in ACCM-2, confirming its tolerance to supraphysiological concentrations of iron (Fig. S2b). To determine the extent to which citrate inhibits C. burnetii iron acquisition, APCM^−FeSO4^ was supplemented with citric acid. C. burnetii replication was significantly impaired on day 4 in the presence of 6 to 7 mM citric acid (Fig. 2a). However, even at the highest concentration of citric acid, C. burnetii replication recovered by day 8 (data not shown). C. burnetii growth deficits in APCM containing 7 mM citric acid could be rescued by additional supplementation of FeSO_4_ (Fig. 2b), indicating that citrate readily complexes with iron.

10.1128/mSphere.00458-20.2 FIG S2 C. burnetii replication in APCM. C. burnetii replication kinetics in APCM^−FeSO4^ and medium supplemented with 5 or 10 μM FeSO_4_ was determined by measuring absorbance every 2 days for a total of 8 days (a). Each point is the average from 3 independent experiments; error bars indicate SEM. C. burnetii was grown axenically in APCM containing different concentrations of FeSO_4_ (b). Bacterial yields were measured after 8 days via GE. Each bar represents the average from 2 independent experiments; error bars indicate SEM. Download FIG S2, PDF file, 0.07 MB. Copyright © 2020 Sanchez and Omsland. 2020 Sanchez and Omsland This content is distributed under the terms of the Creative Commons Attribution 4.0 International license.

Many pathogenic bacteria employ specific mechanisms to acquire physiologically relevant iron-citrate complexes (29, 30). To confirm that our axenic media indeed contained iron-citrate complexes, we analyzed the buffers of both ACCM-2 and APCM against solutions of iron chloride, citric acid, or a 1:1 mixture of iron chloride and citric acid by UV-visible spectroscopy. For these analyses, 0.25 mM solutions were analyzed by spectroscopy from 200 to 500 nm, as described previously (31). As expected (31), citric acid does not absorb beyond 230 nm, iron chloride exhibits strong absorbance at ∼300 nm, and iron chloride:citric acid exhibits a smooth absorbance profile (Fig. 2c). The spectrum obtained for ACCM-2 buffer showed a profile similar to that obtained with iron chloride:citric acid (Fig. 2d). In comparison, the spectrum for APCM buffer is unique compared to the stock solutions and ACCM-2 buffer but indicated an absence of iron-citrate complexes (Fig. 2d). These data illustrate that in ACCM-2, iron-citrate complexes represent a dominant chemical species. Overall, C. burnetii appears incapable of effectively utilizing iron complexed to citrate.

### C. burnetii exhibits poor iron-dependent growth capacity relative to other Gram-negative bacteria.

**FIG 3:**
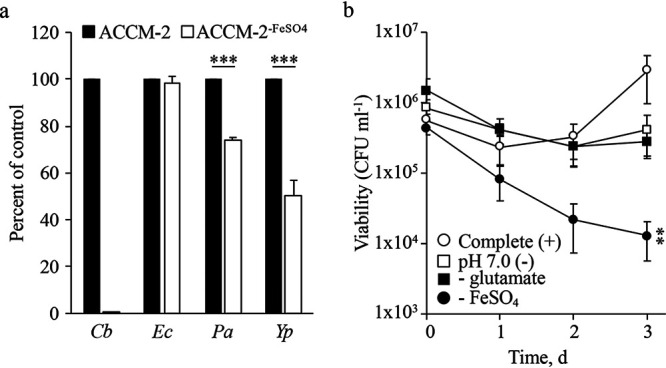
C. burnetii has a poor capacity to replicate in citrate-based medium without iron supplementation. To assess whether iron-dependent growth in ACCM-2 was specific to C. burnetii, cultures of E. coli (*Ec*), Y. pestis (*Yp*), and P. aeruginosa (*Pa*) were compared to that of C. burnetii (*Cb*) after incubation in ACCM-2 or ACCM-2^−FeSO4^ (a). Final culture turbidities were quantified via absorbance and presented as percentage of the control (i.e., ACCM-2 final yields). Each bar represents the average from 3 independent experiments, and error bars indicate SEM. ***, *P* < 0.0001 (unpaired Student’s *t* test versus control conditions). The significance of iron compared to other nutritional or physicochemical conditions established as critical for optimal C. burnetii growth was determined by enumerating CFUs for the initial 3 days of culture where a single component or parameter was missing and/or altered (b). Each point represents the average from 3 independent experiments, and error bars indicate SEM. **, *P* < 0.01 (unpaired Student’s *t* test of starting versus final CFU).

The data demonstrating that C. burnetii is dependent on >5 μM FeSO_4_ in ACCM-2 for optimal replication led to the question of whether this was specific to C. burnetii. Therefore, final culture yields of C. burnetii, Escherichia coli, Pseudomonas aeruginosa, and Yersinia pestis cultured in ACCM-2 or ACCM-2^−FeSO4^ were compared. Inocula for all organisms were 1 × 10^6^ CFU ml^−1^, and for bacteria other than C. burnetii medium pH was adjusted to 7.4. Apart from C. burnetii, all bacteria tested had the ability to replicate in ACCM-2^−FeSO4^, but P. aeruginosa and Y. pestis had an ∼25 to 50% reduction in final yields, respectively, compared to complete ACCM-2 (i.e., optical density at 600 nm for P. aeruginosa [OD_600nm_ *_Pa_*] = 1.88 versus 1.38; OD_600nm_ for Y. pestis [OD_600nm_ *_Yp_*] = 0.41 versus 0.18), while yields obtained with E. coli were indistinguishable from the positive control (Fig. 3a). Additionally, E. coli growth was assessed under conditions matching those for C. burnetii to eliminate moderately acidic pH, 5% O_2_, and 5% CO_2_ as confounding factors, but no change in outcome was observed (Fig. S3). These results suggest that compared to other Gram-negative bacteria, C. burnetii has a reduced capacity to acquire iron.

10.1128/mSphere.00458-20.3 FIG S3 E. coli growth in ACCM-2^−FeSO4^, ACCM-2, and FeSO_4_ gradient under microaerobic conditions. As a direct comparison to culture conditions used for C. burnetii, E. coli was cultured in ACCM-2 and ACCM-2^−FeSO4^ at pH 4.75 under ambient air (a) or in the presence of 5% CO_2_ and O_2_ (b). Each bar represents the average from 2 to 3 independent experiments; error bars indicate SEM. Download FIG S3, PDF file, 0.07 MB. Copyright © 2020 Sanchez and Omsland. 2020 Sanchez and Omsland This content is distributed under the terms of the Creative Commons Attribution 4.0 International license.

To test the relative significance of iron in maintenance of C. burnetii viability, CFU assays were performed. For these assays, the chemically defined (citrate-based) axenic medium D-ACM (26, 32) was used, allowing for controlled nutrient conditions. In complete D-ACM C. burnetii replicated as expected with CFUs increasing over the 3-day incubation (Fig. 3b). In the absence of iron, an ∼2-log loss in viability was observed after 3 days (Fig. 3b). In contrast, C. burnetii cultured without glutamate, a primary carbon/energy source of C. burnetii (28, 33, 34) (Fig. 3b), or at nonpermissive pH 7.0 (Fig. 3b) did not appear to replicate but remained viable. These data suggest that C. burnetii viability is more dependent on iron than on the availability of a primary carbon source or moderately acidic pH, which is required to activate C. burnetii metabolism (28, 33).

### Iron availability differentiates C. burnetii replication from protein and ATP synthesis.

**FIG 4:**
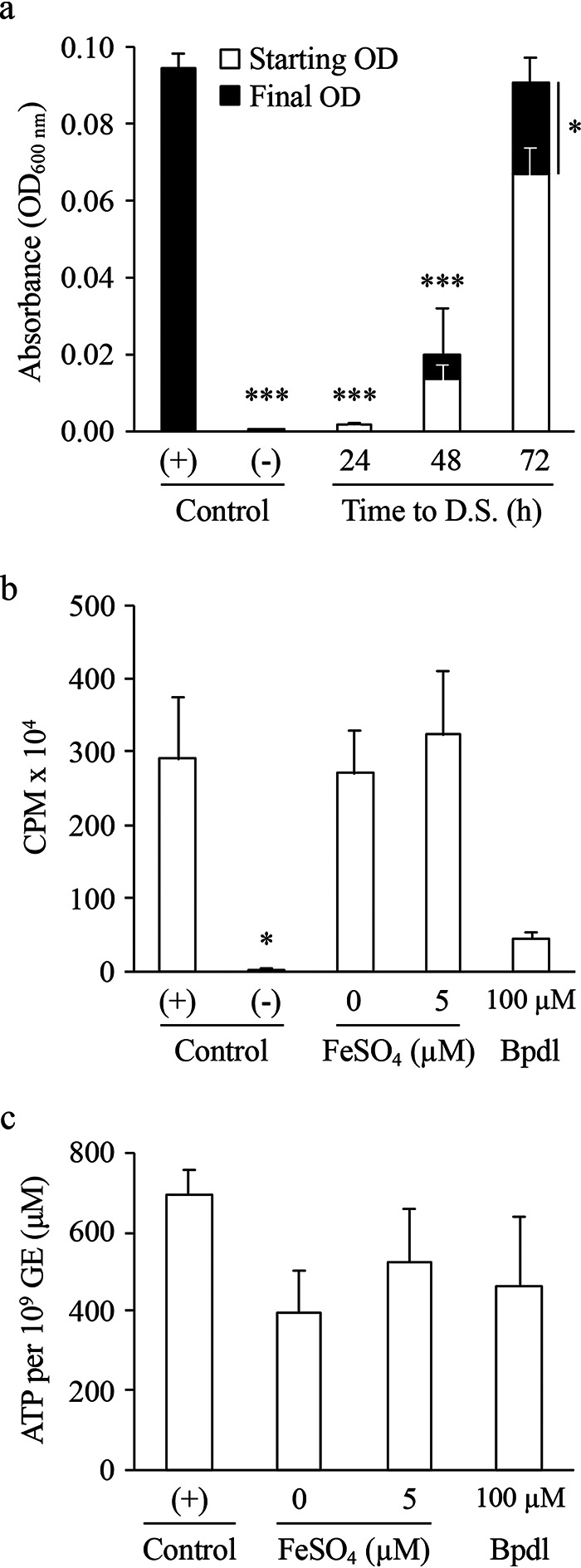
C. burnetii protein and ATP synthesis are permissive to suboptimal iron availability. The requirement for iron to initiate and/or sustain C. burnetii replication and metabolism was assessed by performing downshift (D.S.) experiments where at 24, 48, and 72 h, C. burnetii cultures grown under optimal iron conditions were subcultured into ACCM-2^−FeSO4^, and final yields were measured by absorbance after 8 days of incubation (a), and by quantifying levels of protein synthesis in C. burnetii cultures containing suboptimal concentrations of iron or supplemented with Bpdl (b). The negative control for protein synthesis (Control -) was ACCM-2, pH 7.0. To correlate energy requirements of replication and protein synthesis with iron availability, bacterial ATP pools were measured following incubation with suboptimal levels of iron or supplementation of Bpdl to the medium (c). All conditions were compared to ACCM-2 (Control +). Bars represent the average from 3 to 5 independent experiments. Error bars indicate SEM. *, *P* < 0.05; ***, *P* < 0.0001 (one-way ANOVA with Dunnett’s posttest applied only to panels a and b; unpaired Student’s *t* test used for starting versus final OD in panel a).

The replication phenotypes observed for C. burnetii upon titration of iron (Fig. 1a) motivated assessment of concentration-dependent effects of FeSO_4_ in key aspects of C. burnetii metabolism. First, we probed the requirement of iron to initiate and sustain C. burnetii replication. Regular ACCM-2 was inoculated with C. burnetii at 1 × 10^7^ GE ml^−1^ to allow a 1:10 subculture of 1 × 10^6^ GE ml^−1^ into ACCM-2^−FeSO4^ at 24, 48, and 72 h (referred to as downshift [D.S.]). Bacteria cultured in regular ACCM-2 or ACCM-2^−FeSO4^ were used as the positive or negative controls, respectively. Absorbance was measured at the start of D.S. and on day 8. Compared to the positive control, C. burnetii cultures that underwent downshifts at 24 h were unable to initiate and/or sustain replication, resulting in significantly lower final yields (Fig. 4a). In comparison, cultures that were downshifted to ACCM-2^−FeSO4^ at 48 h were able to initiate but not sustain replication (Fig. 4a). Cultures downshifted at 72 h, however, were capable of overcoming the iron limitation during the last 5 days of culture and reached final yields that were significantly different from starting turbidities (i.e., day 3) and comparable to that of the positive control on day 8 (Fig. 4a). Thus, iron is required to initiate and sustain C. burnetii replication in this axenic model.

In addition to replication, we assessed the requirement of iron in protein synthesis. These experiments were designed to test metabolic fitness as measured by [^35^S]cysteine-methionine ([^35^S]Cys-Met) incorporation into C. burnetii total proteins (35), following a preincubation under different iron availability. Therefore, ∼1 × 10^9^ GE of C. burnetii was incubated in 0.5 ml of regular ACCM-2 (+ control), ACCM-2 at pH 7.0 (− control), ACCM-2 supplemented with 5 μM FeSO_4_, ACCM-2^−FeSO4^ (0 μM FeSO_4_), or ACCM-2 supplemented with 100 μM iron chelator bipyridyl (Bpdl) for 24 h and then transferred to a labeling medium, and the amount of incorporated [^35^S]Cys-Met was measured after 3 h. Bacteria incubated under positive control conditions had high relative incorporation of [^35^S]Cys-Met and vice versa under negative control conditions (Fig. 4b). In contrast to what was observed for analysis of C. burnetii replication, suboptimal levels of FeSO_4_ (i.e., both 0 and 5 μM FeSO_4_) did not correlate with a reduction in [^35^S]Cys-Met incorporation (Fig. 4b). Nevertheless, incorporation of [^35^S]Cys-Met was reduced in the presence of 100 μM Bpdl to levels similar to the negative control; however, this trend was not statistically significant (*P* = 0.0694) (Fig. 4b). Overall, these data show that sustained C. burnetii protein synthesis requires less iron than sustained axenic replication, suggesting a role for iron in triggering pathogen replication.

Iron serves as a cofactor for molecules critical in energy metabolism. Therefore, the possibility that iron limitation could negatively affect C. burnetii synthesis of ATP—for example, to establish an energy charge suitable for replication (34)—was tested. Determination of relative ATP pools was performed by subculturing log-phase (i.e., 3-day) bacteria in ACCM-2 into ACCM-2^−FeSO4^ (0 μM), ACCM-2 containing 5 μM FeSO_4_, or ACCM-2 containing 100 μM Bpdl. Conditions were maintained for 48 h before the relative bacterial ATP pools were quantified. Similar to data obtained for protein synthesis, there was no significant difference between control cultures and bacteria incubated with suboptimal levels of iron (i.e., both 0 and 5 μM FeSO_4_) (Fig. 4c). While there was less relative ATP in bacteria incubated with 100 μM Bpdl compared to the positive control, the difference was not significant. Therefore, suboptimal ATP pools do not explain the discrepancy observed between C. burnetii replication and iron availability.

### C. burnetii exhibits differential responses to free, bound, or complexed forms of iron.

Current annotation of the C. burnetii genome suggests the bacterium harbors few genes related to iron acquisition, especially for complexed or bound forms of iron. Therefore, we assessed the ability of C. burnetii to utilize sequestered and bound forms of iron during axenic replication. We confirmed that C. burnetii is unable to replicate axenically when iron is sequestered with Bpdl (Fig. S4a), a phenomenon that can be reversed with supplementation of FeSO_4_ at ≥25 μM (Fig. S4b). These data are consistent with C. burnetii dependence on free iron. Moreover, in APCM^−FeSO4^, inhibition of C. burnetii replication occurred with Bpdl when supplemented at a 10-fold-lower concentration than in ACCM-2 cultures (Fig. S4c). Therefore, even in APCM iron must be unsequestered to permit C. burnetii replication. To compare the effects of iron sequestration via Bpdl on C. burnetii replication to that of a different bacterium, E. coli was cultured in ACCM-2^−FeSO4^—conditions permissible to growth of this bacterium—supplemented with Bpdl. Obtained data indicate that E. coli has a greater capacity to replicate when iron is both limited and sequestered (Fig. S4c), compared to C. burnetii.

10.1128/mSphere.00458-20.4 FIG S4 C. burnetii cannot utilize sequestered forms of iron. C. burnetii cultures in ACCM-2-based medium with optimal or suboptimal concentrations of iron were subjected to various concentrations of Bpdl for 8 days before final yields were measured by absorbance (a). To determine whether effects contributed by Bpdl could be rescued with additional iron, ACCM-2 cultures containing 100 μM Bpdl were supplemented with additional FeSO_4_ and C. burnetii final yields were measured via absorbance (b). C. burnetii final yields were measured by absorbance in APCM^−FeSO4^ containing different concentrations of Bpdl (c). Each bar represents the average from 2 to 3 independent experiments; error bars indicate SEM. The effect of Bpdl on E. coli viability was evaluated in ACCM-2^−FeSO4^ containing different concentrations of Bpdl for 24 h (d). Viability was determined at the onset and after Bpdl exposure via CFU enumeration on LB agar. Each data point represents the average from 2 independent experiments; error bars indicate SEM. *, *P* < 0.05; **, *P* < 0.01 (unpaired Student’s *t* test). Download FIG S4, PDF file, 0.08 MB. Copyright © 2020 Sanchez and Omsland. 2020 Sanchez and Omsland This content is distributed under the terms of the Creative Commons Attribution 4.0 International license.

**FIG 5:**
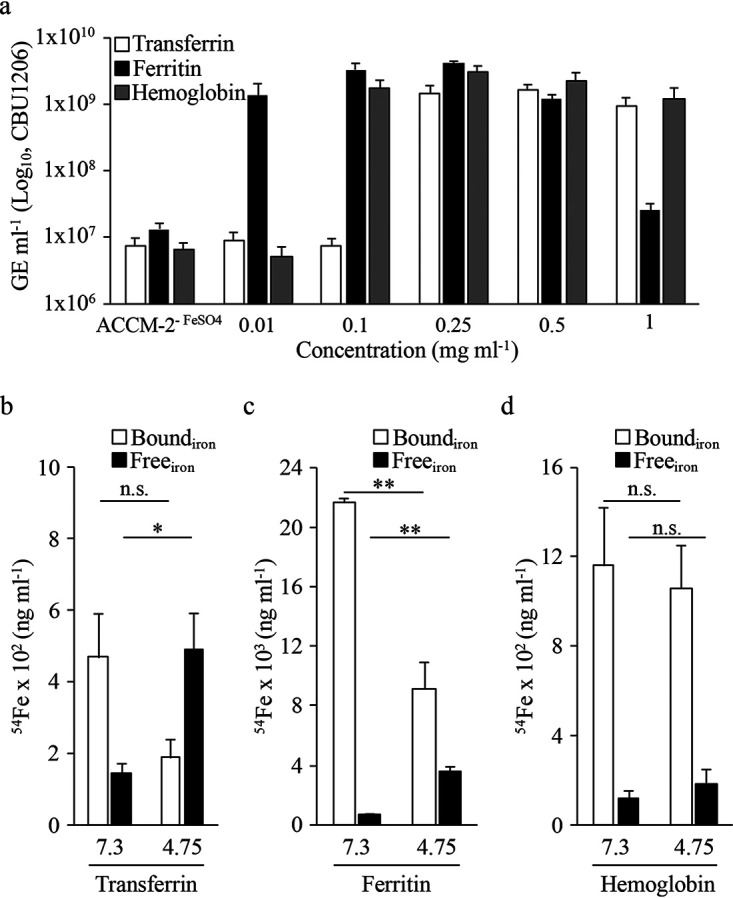
Transferrin, ferritin, and hemoglobin stimulate C. burnetii replication. To assess whether C. burnetii can utilize iron from host-associated iron-containing proteins, bacterial yields were determined via enumeration of GE after 8 days in ACCM-2^−FeSO4^ supplemented with different concentrations of transferrin, ferritin, or hemoglobin (a). Each bar represents the average from 3 independent experiments; error bars indicate SEM. To determine whether the moderately acidic pH of the CCV aids iron release from iron-binding proteins, iron content was measured for 0.5 mg ml^−1^ of transferrin (b), ferritin (c), or hemoglobin (d) exposed to pH 7.3 or 4.75 for 2 h. Each bar represents the average from 3 independent experiments; error bars indicate SEM. n.s., not significant; *, *P* < 0.05; **, *P* < 0.01 (unpaired Student’s *t* test).

During natural infection, C. burnetii likely encounters iron-containing molecules, including ferritin, hemoglobin, and transferrin. To determine whether C. burnetii has the ability to utilize host-associated bound forms of iron, ACCM-2^−FeSO4^ was supplemented with various concentrations of transferrin, ferritin, or hemoglobin, and final yields were measured after 8 days via quantification of GE. Optimal C. burnetii yields were supported by ≥0.25 mg ml^−1^ transferrin and 0.1 to 1 mg ml^−1^ of hemoglobin without toxic effects (Fig. 5a). In comparison, ferritin produced similar trends as molecular iron (Fig. S1b), with maximal yields obtained with 0.01 to 0.25 mg ml^−1^ ferritin and a gradual decrease in final yields as concentrations increased further (Fig. 5a). ICP-MS analysis revealed that iron content in ferritin ranged between ∼9 μM and ∼171 μM at 0.01 mg ml^−1^ and 0.25 mg ml^−1,^ respectively (Fig. S5), which correlates with concentrations of free iron supporting C. burnetii replication.

10.1128/mSphere.00458-20.5 FIG S5 Relative content of molecular iron in 0.01 and 0.25 mg ml^−1^ of transferrin, ferritin, and hemoglobin was quantified via ICP-MS. Each bar represents the average from 2 to 3 independent experiments; error bars indicate SEM. Download FIG S5, PDF file, 0.07 MB. Copyright © 2020 Sanchez and Omsland. 2020 Sanchez and Omsland This content is distributed under the terms of the Creative Commons Attribution 4.0 International license.

It remains unclear whether C. burnetii utilizes the intact transferrin, ferritin, or hemoglobin protein versus just the associated molecular iron, which has been shown to be released from such proteins following exposure to acidic conditions. Because the CCV lumen is mildly acidic (i.e., pH 4.5 to 5.5) and C. burnetii appears to exhibit preference for molecular iron, we tested whether a moderately acidic pH induces iron release from transferrin, ferritin, and hemoglobin. For these analyses, 0.5 mg ml^−1^ samples of transferrin, ferritin, or hemoglobin were incubated in ACCM-2 inorganic basal buffer (26) (without FeSO_4_) adjusted to pH 7.3 (i.e., cytosolic pH) or 4.75 (i.e., CCV pH) for 2 h at 37°C and then analyzed by ICP-MS to measure iron content. For transferrin and ferritin, the relative concentration of free and/or released iron following incubation increased significantly at pH 4.75 versus 7.3, with a corresponding loss in bound iron (Fig. 5b). For hemoglobin, there was no apparent difference in the concentration of bound or free iron regardless of the pH used, suggesting that the pH values tested were unable to induce release of iron within 2 h. It should be noted that iron recycling from hemoglobin involves enzyme-dependent (heme oxygenase) degradation within erythrophagocytic macrophages (36). Nevertheless, these results indicate that for ferritin and transferrin the mildly acidic pH associated with the CCV induces iron release from these proteins.

### Host intracellular iron potentiates C. burnetii intracellular replication.

**FIG 6:**
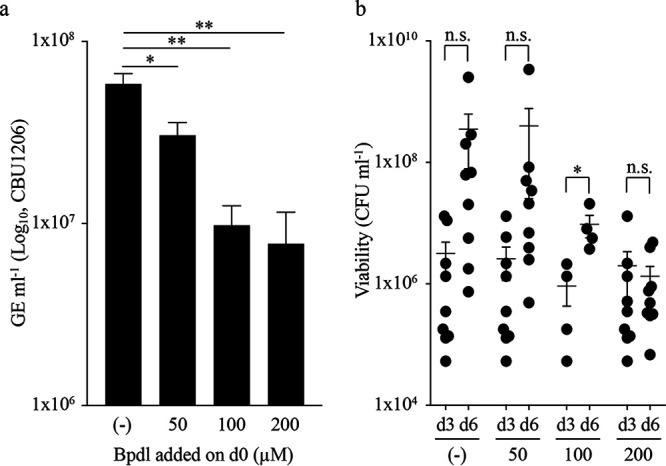
Modulation of host intracellular iron content directly influences C. burnetii intracellular replication. The influence of host iron pools on C. burnetii intracellular replication was assessed by exposing infected Vero cells for 3 days to different concentrations of Bpdl at onset of infection and measuring C. burnetii load via GE (a). Each bar represents the average of 2 to 6 independent experiments; error bars indicate SEM. *, *P* < 0.05; **, *P* < 0.01 (one-way ANOVA with Dunnett’s posttest). C. burnetii intracellular viability was determined via CFU enumeration from Vero cells exposed to Bpdl for 3 days following addition on day 3 (d3) postinfection (b). Data points indicate biological replicates with the mean and SEM represented. n.s., not significant; *, *P* < 0.05 (paired Student’s *t* test).

Due to the intracellular nature of C. burnetii, we assessed whether modulation of host iron pools alters pathogen replication in cultured cells. Vero cells were infected with C. burnetii constitutively expressing green fluorescent protein (GFP) and incubated for 3 days using cell culture media containing 50, 100, and 200 μM Bpdl; incubation without Bpdl added to the medium served as the positive control. C. burnetii replication decreased significantly after 3 days with increasing Bpdl concentrations, compared to non-Bpdl-treated cells (Fig. 6a). Additionally, C. burnetii intracellular viability was determined using the aforementioned Bpdl concentrations. Bpdl, however, was added on day 0 (d0) or 3 (d3), and infections were maintained for an additional 3 days, with C. burnetii intracellular viability quantified at onset and end of Bpdl treatment. When ≥50 μM Bpdl was added on d0, C. burnetii d3 intracellular CFUs trended but were not significantly lower than d0 CFUs (data not shown). In comparison, only 200 μM Bpdl added on d3 resulted in an overall lower mean value for final CFUs on d6, but again differences were not statistically significant (Fig. 6b). These trends were corroborated in representative micrographs where GFP puncta, indicative of replicating C. burnetii, were reduced in the presence of 50 to 200 μM Bpdl, depending on onset of chelator supplementation (Fig. S6). Bright-field images show that host cells maintain their integrity under the Bpdl conditions used (Fig. S6). These data indicate that, similarly to results obtained from axenic culture, C. burnetii is dependent on host free iron for replication. Furthermore, the data suggest that C. burnetii in part relies on the host labile iron pool (LIP), as Bpdl has been shown to specifically affect the LIP of eukaryotic cells (37, 38).

10.1128/mSphere.00458-20.6 FIG S6 C. burnetii intracellular replication in the presence of Bpdl. Representative end-point bright-field (BF) and fluorescence (GFP) micrographs of Vero cells infected with GFP-expressing C. burnetii and exposed to different concentrations of Bpdl at 0 (a) and 3 (b) days postinfection. Download FIG S6, PDF file, 0.4 MB. Copyright © 2020 Sanchez and Omsland. 2020 Sanchez and Omsland This content is distributed under the terms of the Creative Commons Attribution 4.0 International license.

## DISCUSSION

Motivated by the tropism of C. burnetii for organs involved in iron storage and recycling, we established an axenic culture model to study the effects of iron on C. burnetii physiology. Use of the citrate-based medium ACCM-2 allowed analysis of C. burnetii responses to iron without use of additional chemical chelators known to also sequester transition metals other than iron with significance in bacterial physiology (40, 41). We demonstrate that C. burnetii replication can occur over a wide concentration range of iron and that C. burnetii likely requires molecular iron for efficient uptake and utilization. Moreover, C. burnetii capacity to grow under iron-limiting conditions is inferior to that of prominent Gram-negative bacteria (i.e., E. coli, P. aeruginosa, and Y. pestis) known to colonize nutritionally diverse niches, consistent with unique adaptation of C. burnetii to a specific niche characterized by availability of molecular iron. Additionally, optimal pathogen protein and ATP synthesis occurs under iron availability that does not support C. burnetii growth. Finally, active sequestration of iron by addition of the membrane-permeant iron chelator Bpdl reduced C. burnetii replication during infection of cultured cells, suggesting C. burnetii partially relies on the host LIP.

With few exceptions, including Borrelia burgdorferi (42), iron is a critical micronutrient for bacterial pathogens. Iron can affect regulation of virulence by triggering virulence factor expression (43–45) and is a cofactor in macromolecules of central metabolism, specifically the tricarboxylic acid (TCA) cycle and oxidative phosphorylation (46). In C. burnetii, iron has been described to play a limited role in virulence regulation, and C. burnetii-induced pathogenesis is actually reduced under conditions of elevated iron, as determined by infection of mice restricted to diets designed to control iron intake (23). Briggs et al. (23) attributed these effects of iron on C. burnetii in part to the potentially toxic effects of iron through Fenton chemistry, specifically reactive oxygen intermediates. As the CCV is fed via fluid phase endocytosis, it is conceivable that in tissues with a high iron load, such as the liver and splenic red pulp, the CCV lumen could accumulate micromolar concentrations of iron. Based on our assessment, C. burnetii appears tolerant to increasing concentrations of molecular iron as there is no significant reduction in bacterial yields in the presence of up to 1 mM FeSO_4_ (see Fig. S1b in the supplemental material).

Certain bacterial pathogens take advantage of and/or adapt to niches characterized by unusually high levels of iron in order to support optimal replication and virulence. For instance, infection with Vibrio vulnificus (47, 48), Yersinia enterocolitica (49, 50), and Listeria monocytogenes (51) results in higher mortality rates in individuals with hereditary hemochromatosis or other iron overload conditions (52). Additionally, some vector-borne bacterial pathogens appear to have adapted to the iron load in the blood meals of their respective vector hosts. These include Bartonella quintana, the louse-transmitted agent of trench fever, which has one of the highest requirements for exogenous heme (53, 54), and the agent of anaplasmosis, Anaplasma marginale, which replicates within mammalian erythrocytes (55). Therefore, it is plausible that the apparent ability to withstand toxicity associated with supraphysiological levels of iron is an evolutionary adaptation of C. burnetii to blood-feeding vectors (e.g., ticks) and organs in mammalian hosts associated with iron storage and recycling. Of potential significance, C. burnetii harbors the gene *ght* (CBU0530), which in Neisseria meningitidis confers resistance to heme iron toxicity (56). It is possible that CBU0530 represents an evolutionary adaptation for C. burnetii resistance to iron-induced stress during colonization of niches with elevated iron. Alternatively, rather than having an unusually high tolerance for iron in order to sustain metabolic processes, C. burnetii may simply have a poor capacity for iron uptake and thus relies on elevated iron availability in order to acquire sufficient amounts for activity. Apparent absence of redundancy in mechanisms for acquisition of iron in C. burnetii is consistent with low efficiency in iron uptake.

The core metabolic machinery of C. burnetii is largely intact. Amino acids alone, some of which funnel into the TCA cycle where iron serves as a primary cofactor for some enzymes, can support C. burnetii replication (32). ATP synthesis via oxidative phosphorylation, a process greatly reduced by chemical inhibition in C. burnetii (34), is another key process dependent on iron as a cofactor. While we show that ATP pools were not significantly reduced following a 48-h period under suboptimal iron conditions, there was an overall trend for reduced ATP when iron was sequestered using Bpdl (Fig. 4c). These observations were similar to that for overall protein synthesis with no significant difference observed under conditions of optimal versus suboptimal iron availability or iron sequestration (Fig. 4b). Therefore, it appears that C. burnetii ATP and protein synthesis are permissive to iron conditions that are insufficient for replication of C. burnetii. Nevertheless, the addition of the iron chelator Bpdl resulted in a reduction in overall protein and ATP levels, suggesting that iron availability directly influences C. burnetii metabolism. While some organisms (e.g., nonpathogenic *Lactobacillus* spp. [57]) are capable of utilizing an alternative cofactor such as manganese in place of iron, C. burnetii replication could not be rescued by manganese (data not shown).

With notable exceptions, including bacteria of the family *Chlamydiaceae* (75), bacterial pathogens can employ several distinct mechanisms to ensure uptake of sufficient levels of iron for replication, even if the extracellular environment contains only nanomolar concentrations of iron. Analysis of the C. burnetii genome has revealed few such mechanisms, although a predicted ferrous iron transporter (FeoAB; CBU1766-1767) is encoded (23), and the protein LimB has been identified as a surface exposed iron-binding protein in this pathogen (76). Therefore, it is expected that the CCV contains molecular iron and that the bacterium is limited in its ability to utilize other forms of iron. Maintenance of iron uptake systems would be crucial for C. burnetii biological success and could explain the current absence of a C. burnetii *feoAB* mutant within current transposon libraries (58–60). Using both citrate-based (ACCM-2) and phosphate-based (APCM) axenic media, we determined that, indeed, C. burnetii is limited in its ability to acquire sequestered (Fig. S4) and citrate-bound (Fig. 2) forms of iron. These data are consistent with the inability of C. burnetii to acquire such iron species via—for example—a TonB-like protein, which would be required for active uptake of siderophores (61, 62) and ferric citrate complexes (63, 64). When we evaluated the oxidation state of iron in ACCM-2 and APCM, Fe^3+^ was the predominate form (Fig. S7a). Analysis of iron oxidation state was also done using extracts from Vero and J774A.1 host cells, revealing that Vero cells predominantly contain ferrous iron whereas in J774A.1 cells Fe^2+^ and Fe^3+^ are present at similar levels (Fig. S7b). These data suggest that ferric iron is an important iron source for C. burnetii, both axenically and in the context of the host. Consideration of Fe^2+^ as a relevant oxidation state is highlighted by evidence suggesting that C. burnetii, in part, relies on the host LIP, as demonstrated by reduced replication in host cells treated with Bpdl (Fig. 6). The eukaryotic cytoplasmic LIP accounts for ∼20% of iron within eukaryotic cells and is composed of chelatable Fe^2+^ (37, 38). Nevertheless, since the CCV is fed by fluid phase endocytosis, it is equally likely that Bpdl directly sequesters ferrous iron within the CCV lumen. Overall, both ferric and ferrous iron may be utilized by C. burnetii during intracellular growth.

10.1128/mSphere.00458-20.7 FIG S7 Iron oxidation states were determined for ACCM-2 and APCM containing 100 μM FeSO_4_ at 0 and 3 days postincubation under microaerobic conditions (a) or in Vero and J774A.1 host cells (b). Each bar represents the average from 3 to 6 independent experiments; error bars indicate SEM. Download FIG S7, PDF file, 0.07 MB. Copyright © 2020 Sanchez and Omsland. 2020 Sanchez and Omsland This content is distributed under the terms of the Creative Commons Attribution 4.0 International license.

Despite the lack of annotated receptors for host-associated iron-containing proteins, C. burnetii exhibited comparable growth kinetics during axenic culture with specific iron-containing proteins or molecular iron (Fig. 5a). This is perhaps not surprising as we demonstrate that the mildly acidic pH associated with the CCV releases molecular iron from transferrin (Fig. 5b) and ferritin (Fig. 5c), but not hemoglobin (Fig. 5d), within 2 h. We hypothesize, however, that during prolonged exposure to CCV pH and degradative enzymes iron will be released also from hemoglobin. Nonetheless, as iron sources, both ferritin and transferrin are expected to be more biologically relevant, since these host-associated proteins are delivered/reside within host cells and, for at least transferrin (24), have been linked with C. burnetii intracellular replication. Poor replication by *C. burnetii* in medium supplemented with hemin suggests *C. burnetii* cannot transport heme (77).

During infection, key mammalian innate immune response factors (e.g., interferon gamma, tumor necrosis factor alpha, interleukin 1 [IL-1], and IL-6 [65, 66]) have evolved to modulate iron metabolism in order to inhibit overall iron release and thus starve invading pathogens for the molecule. In response, many bacterial pathogens, including Y. pestis (67) and P. aeruginosa (45), use low iron levels to initiate virulence factor expression via the Fur transcription factor. It has been shown that while C. burnetii encodes elements of the Fur regulon, there are limited Fur-regulated genes (23) and therefore C. burnetii is expected to have a limited ability to respond to iron availability. Rickettsia rickettsii also appears limited in its capacity to respond to iron availability in terms of regulation but still undergoes growth arrests (69), consistent with iron exhibiting metabolic rather than regulatory growth inhibition. Ellison et al. suggested that iron concentrations within the replicative niche (i.e., cytoplasm) of R. rickettsii may be more consistent, thus limiting the need to respond to iron limitations via transcriptional changes (69). Therefore, in environments where iron availability is consistently limited, it can be expected that bacterial iron acquisition systems be robust and controlled via Fur or similar regulation. However, for environments where iron is sufficiently available and in a labile form, redundant acquisition systems and transcription-based regulation could prove nonessential for bacteria.

### Conclusions.

**FIG 7:**
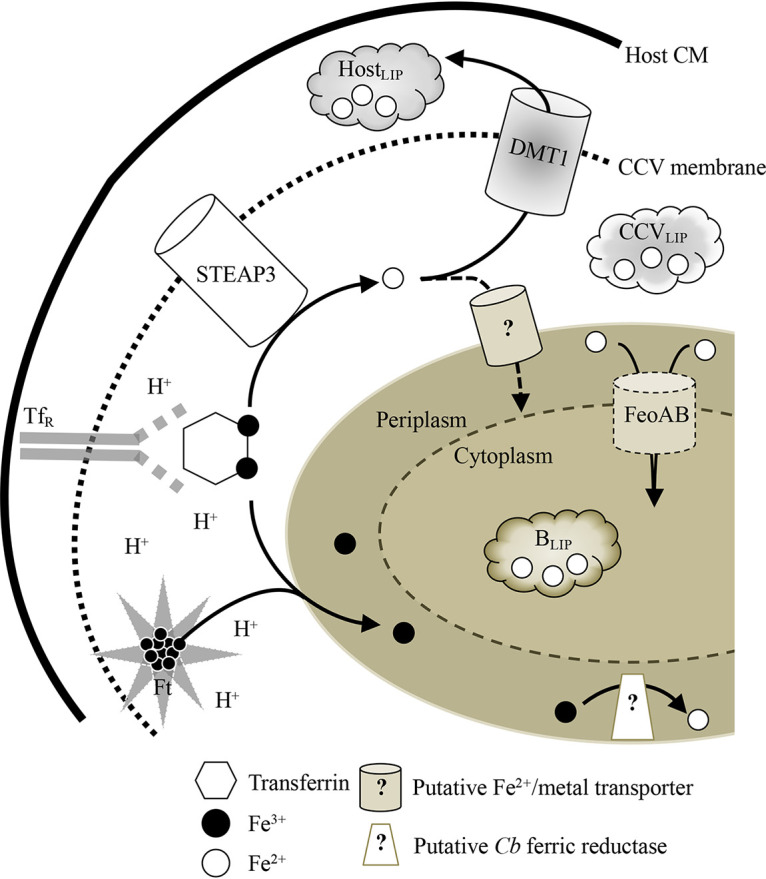
Working model for the role of iron in C. burnetii replication and viability. C. burnetii acquires molecular iron through acid degradation of iron-binding proteins (e.g., transferrin [Tf] and ferritin [Ft]) after uptake into the acidic CCV. Ferric iron released from iron-containing proteins is likely reduced within the CCV via the host enzyme STEAP3, delivered to the CCV upon fusion with endosomes. Transportation of Fe^3+^ into the periplasm of *Coxiella* would require conversion of Fe^3+^ to Fe^2+^ via a *Coxiella*-specific ferric reductase in order for C. burnetii to transport Fe^2+^ via FeoAB to the cytosol for replication and viability. Alternatively, Fe^3+^ reduced via STEAP3 to Fe^2+^ can be (i) shuttled outside the CCV via the DMT1 transporter and maintained in the host labile iron pool (Host_LIP_), (ii) remain within the CCV in a similar labile iron pool (CCV_LIP_), or (iii) be actively transported into the *Coxiella* periplasm by a putative Fe^2+^/metal transporter. Once in the periplasmic space, Fe^2+^ can be directly acquired via FeoAB for use in bacterial replication and viability or reside within a putative bacterial labile iron pool (B_LIP_).

The requirement for molecular iron by C. burnetii in supporting optimal replication and viability is consistent with genome sequence analysis suggesting iron uptake occurs via the FeoAB transporter. Figure 7 illustrates our current working model of the iron acquisition strategies employed by C. burnetii. The moderately acidic and degradative properties of the CCV likely support active degradation and release of iron from iron-containing proteins in the mammalian body. This is supported by demonstrated release of iron from transferrin (7), ferritin (70, 71), and hemoglobin (72) upon exposure to moderately acidic pH, reactive oxygen species, and/or degradative enzymes—all of which characterize the CCV (18, 19). Once iron is released from iron-containing host proteins, it remains unclear how C. burnetii acquires molecular iron and, more importantly, which oxidation state of iron is available to the bacterium. It is well established that within endosomes (known to fuse with the CCV) upon degradation of transferrin, Fe^3+^ is released, reduced by STEAP3 to Fe^2+^, and then shuttled into the host cell cytoplasm via the transporter DMT1 (1). Fe^2+^ then resides in the label iron pool (LIP) or is stored bound to ferritin (Ft). Fe^3+^ appears to be the predominate iron species in axenic cultures and Vero cells but equally prevalent as Fe^2+^ in J774A.1 cells (Fig. S7). Therefore, within the CCV C. burnetii may have access to both Fe^3+^ and Fe^2+^.

## MATERIALS AND METHODS

### Bacteria and culture conditions.

C. burnetii Nine Mile phase II (NMII; RSA 439, clone 4) was propagated in ACCM-2 and prepared for long-term storage as described previously (26, 27). Culture inocula were normalized to GE (26). C. burnetii was cultured in the chemically complex ACCM-2 (26, 27), chemically defined D-ACM (26, 30), and newly formulated acidified phosphate *Coxiella* medium (APCM), pH 4.75, with the following chemical composition: 49.9 mM KH_2_PO_4_, 152 mM KCl, 59.9 mM NaCl, 1.96 mM MgCl_2_·6H_2_O, 0.007 mM CaCl_2_·2H_2_O, 2.5 mg/ml Casamino Acids, 1.5 mM l-cysteine hydrochloride, 0.1 mg/ml peptone, 1.0 mg/ml methyl-β-cyclodextrin, and 12.5% (vol/vol) RPMI 1640 without l-glutamine, supplemented with GlutaMAX (Gibco BioSciences, Dublin, Ireland). Liquid cultures were established in T-25, 6-well, or 12-well polystyrene cell culture flasks/plates containing 7, 3, or 1.5 ml of medium, respectively, and inoculated with ∼1 × 10^6^ GE ml^−1^ bacteria, unless otherwise noted. ACCM-2 supplemented with tryptophan, pH 4.5 (26), was used for CFU analysis. Liquid and solid C. burnetii cultures were maintained for 7 to 8 days at 37°C with 5% CO_2_ and 5% O_2_. For visualization of C. burnetii during host cell infection, a strain expressing green fluorescent protein (GFP) (C. burnetii NMII Tn7-CAT-GFP) was used (78). The pH of ACCM-2 was adjusted to ∼7.4 when culturing E. coli, P. aeruginosa, and Y. pestis in the medium. Starter cultures for E. coli MG1655 and P. aeruginosa were prepared in Luria-Bertani (LB) broth and grown at 37°C with shaking (∼200 rpm) overnight. Starter cultures for Y. pestis KIM6+ were prepared in heart infusion broth (HIB) at 25 to 30°C with shaking (∼200 rpm) overnight.

### Eukaryotic cell culture and infection.

Mouse macrophage-like (J774A.1) cells (TIB-67; ATCC) and African green monkey kidney (Vero) cells (CCL-81; ATCC) were maintained in complete RPMI 1640 medium (i.e., RPMI 1640 without l-glutamine [Corning Cellgro; Corning Inc., Corning, NY], supplemented with GlutaMAX [Gibco BioSciences, Dublin, Ireland] and 5% [vol/vol] heat-inactivated serum complex [hi-FetalPlex; Gemini Bio-Products, West Sacramento, CA]) at 37°C and 5% CO_2_. For infections, host cells were seeded in 12-well plates at a density of 2 × 10^5^ cells per 1.5 ml and maintained in complete RPMI 1640 at 37°C and 5% CO_2_ for 24 h. Subsequently, host cells were infected with 1 × 10^7^ GE ml^−1^ of bacteria in 1 ml of plain RPMI 1640 (i.e., RPMI 1640 without l-glutamine supplemented with only GlutaMAX). Infections were facilitated by centrifugation at 400 × *g* for 30 min at room temperature (RT). Following infection, inocula were removed and host cells were washed once with plain RPMI 1640 to remove noninternalized bacteria. Infected cells were maintained in 1.5 ml of RPMI 1640 (Corning Cellgro; Corning Inc., Corning, NY) containing 2% (vol/vol) hi-FetalPlex and incubated at 37°C and 5% CO_2_.

### Radiolabeling with [^35^S]cysteine-methionine.

Radiolabeling of C. burnetii proteins was performed essentially as described previously (73). Briefly, ∼1 × 10^9^ GE of stock organisms was preincubated in 0.5 ml of ACCM-2^−FeSO4^ or ACCM-2 containing 5 μM FeSO_4_, 10 μM FeSO_4_, or 100 μM Bpdl for 24 h in 24-well plates. Following preincubation, cultures were pelleted via centrifugation at 20,000 × *g* for 5 min and then washed in citrate labeling buffer without iron (26.5 mM citric acid, 32.2 mM tribasic sodium citrate, 50 mM glycine, 5 mM glutamate, 1 mM glucose, 42.8 mM NaCl, 3.7 mM KH_2_PO_4_, 2.7 mM KCl, 1.0 MgCl_2_, 0.1 mM CaCl_2_, pH 4.5). Bacteria were then resuspended in 0.5 ml of citrate labeling buffer that contained the same concentration of iron sulfate to which the cells were exposed during the preincubation. To allow incorporation of radiolabel into proteins, 10 μl EasyTag Express protein labeling mix (i.e., [^35^S]Cys-Met) was added directly to each cell suspension, and cells were allowed to incubate for 3 h at 37°C and 5% CO_2_. Following incubation, cells were washed with 0.2 ml of sodium phosphate saline (10 mM Na_2_PO_4_, 10 mM NaH_2_PO_4_, 150 mM NaCl, pH 7.8) and then lysed with 50 μl lysis buffer (87.5 mM Tris-HCl [pH 6.8], 89.7 mM SDS, 350 mM β-mercaptoethanol, 38 μM bromophenol blue, 9% glycerol) and heated to 95 to 100°C for 10 min. Equal volumes of each sample were analyzed via scintillation counting to determine counts per minute (cpm). Use of radioactive materials was approved by the Radiation Safety Officer, Washington State University.

### Determination of bacterial ATP pools.

Quantification of bacterial ATP pools was performed using an ATP determination kit (ThermoFisher Scientific, Waltham, MA). Briefly, ACCM-2 cultures were inoculated with ∼1 × 10^6^ GE ml^−1^ C. burnetii, allowed to replicate for 3 days, and then subcultured into medium containing 1 μM FeSO_4_, 5 μM FeSO_4_, or 100 μM Bpdl. Original cultures and subcultures were allowed to progress for an additional 48 h under 5% CO_2_ and 5% O_2_. At time of sampling, cultures were transferred to prechilled centrifuge tubes and incubated on ice for 3 to 5 min. Bacterial cells were then pelleted via centrifugation (20,000 × *g*, 10 min at 4°C). Supernatants were removed, and cells were washed with 1.0 ml of ACCM-2 inorganic basal buffer (26) before being repelleted. Supernatants were removed, pellets were resuspended in 0.01 ml 0.01% (wt/vol) SDS in sterile ultrapure water (MΩH_2_O; 18.2 MΩ; Milli-Q integral water purification systems; EMD Millipore), and ATP was extracted by heating samples to 95 to 100°C for 5 min. Samples were then transferred to −80°C until analysis. To analyze ATP pools, samples were diluted in 0.09 ml sodium phosphate saline (10 mM Na_2_PO_4_, 10 mM NaH_2_PO_4_, 150 mM NaCl, pH 7.8), and 0.01 ml of this mixture was added to 0.09 ml of kit master mix for analysis following the manufacturer’s instructions. The concentration of ATP was extrapolated from a standard curve and normalized to GE determined at time of sampling.

### Determination of acid-induced iron release from hemoglobin, ferritin, and transferrin.

To measure pH-dependent release of iron from iron-binding proteins, 1-ml aliquots of 0.5 mg ml^−1^ holo-hemoglobin, ferritin, and transferrin were dissolved in ACCM-2 inorganic basal buffer (26) without FeSO_4_ at either pH 7.3 or 4.75. These solutions were incubated for 2 h at 37°C before being passed through a 3-kDa filter (Amicon Ultra-0.5 centrifugal filter unit; MilliporeSigma, Burlington, MA) to separate iron bound to intact proteins from the free iron. To process, 0.1 ml concentrated HNO_3_ was added to the filtrates to extract free iron overnight at room temperature, heated to 80°C for 1 h, and then diluted to a final volume of 5 ml with double-distilled water (ddH_2_O). Iron content was measured by inductively coupled plasma mass spectroscopy (ICP-MS) using an Agilent 7700 series ICP-MS instrument (Agilent Technologies, Santa Clara, CA).

### Measuring influence of host iron content on C. burnetii replication and viability.

To analyze the influence of host iron pools on C. burnetii intracellular replication, 0, 50, 100, and 200 μM of the iron chelator Bpdl was added to the culture medium in duplicate wells, infections were allowed to progress for 3 days, and C. burnetii yields were enumerated via quantification of GE. Representative cultures were imaged via fluorescence microscopy using a Leica Dmi8 inverted microscope (Leica Microsystems, Buffalo Grove, IL). To measure the influence of host iron pools on C. burnetii intracellular viability, infections were subjected to 0, 50, 100, and 200 μM Bpdl on day 0 or 3 postinfection in duplicate and infections were allowed to progress for an additional 3 days before viability was enumerated via a CFU assay. Briefly, the medium was removed and 0.25 ml of Trypsin-EDTA (Sigma-Aldrich, St. Louis, MO) was added to detach cells. An 0.75-ml amount of ACCM-2^−FeSO4^, pH 4.75, was then added to the trypsinized host cells and homogenized well using a micropipette before being transferred to a Lysis Matrix H tube (MP Biomedicals, LLC, Irvine, CA). Host cells were subjected to mechanical lysis (FastPrep-24; MP Biomedicals, LLC, Irvine, CA) via two 20-s pulses at 6.0 m s^−1^, and then host debris was pelleted by centrifugation for 10 min at 1,500 rpm. Supernatants were serially diluted, and 10 μl of each dilution was spotted on top solid ACCM-2 supplemented with tryptophan, pH 4.5 (26), and incubated for 7 to 8 days at 37°C with 5% CO_2_ and 5% O_2_.

### Determination of iron content in axenic media and bound forms of iron.

ACCM-2 containing 5, 25, or 50 μM FeSO_4_ was prepared in a total volume of 1 ml. Lyophilized powders were used to generate 1-ml aliquots of 50 and 1,250 μg ml^−1^ bovine hemoglobin (Sigma-Aldrich, St. Louis, MO), bovine transferrin (Sigma-Aldrich, St. Louis, MO), and equine spleen ferritin (Sigma-Aldrich, St. Louis, MO) in MΩH_2_O. To extract iron, 0.1 ml concentrated HNO_3_ was added to each sample and incubated at room temperature overnight. Samples were then heated to 80°C for 1 h before being diluted to 5 ml in ddH_2_O, generating final concentrations of 1, 5, and 10 μM FeSO_4_ for ACCM-2 and APCM, and 10 and 250 μg ml^−1^ for each iron-binding protein. Iron content was measured as described above by ICP-MS.

### Determination of iron oxidation state in axenic media and host cells.

To measure the oxidation state of iron in axenic media, 7 ml of freshly prepared ACCM-2 and APCM containing 100 μM FeSO_4_ was added to T-25 flasks in duplicate and incubated under microaerobic conditions (i.e., 5% O_2_ and 5% CO_2_). At 0 and 3 days postincubation, 50 μl of medium was added to a 96-well plate in triplicate and analyzed for iron oxidation state using the iron assay kit (Sigma-Aldrich, St. Louis, MO). The assay was performed following manufacturer’s instructions. To measure iron oxidation states in host cells, cells were incubated to confluence in 10 T-75 flasks. Vero cells were detached from flasks using Trypsin-EDTA (Sigma-Aldrich, St. Louis, MO), and J774A.1 cells were detached via scraping. Host cells were washed twice with phosphate-buffered saline (PBS; pH 7.4), resuspended in 1 ml of assay buffer, and transferred to Lysis Matrix H tubes (MP Biomedicals, LLC, Irvine, CA). Host cells were lysed via two 20-s pulses at 6.0 m s^−1^ before insoluble host debris was removed by centrifugation for 10 min at 16,000 × *g*, 4°C. The resulting supernatant was used directly. Host cell numbers were determined via direct cell count prior to lysis for normalization purposes.

### Preparation of 2,2′-bipyridyl.

2,2′-Bipyridyl (Bpdl, C_10_H_8_N_2_; Sigma-Aldrich, St. Louis, MO) (79) was dissolved in ethanol and stored at −20°C.

### Quantifying C. burnetii GE.

Quantification of bacteria by genome equivalents (GE) was performed as described previously (26). Briefly, 1 ml of C. burnetii NMII cultures was added to a 1.5-ml screw-cap tube containing 0.1-mm zirconia beads (BioSpec Products, Bartlesville, OK) and subjected to mechanical lysis (FastPrep-24; MP Biomedicals) via three 30-s pulses at 5.0 m s^−1^. Samples were serially diluted, and GE were quantified via detection of the C. burnetii gene CBU1206 (74) using a CFX96 Real-time PCR detection system (Bio-Rad Laboratories, Hercules, CA) and the iTaq Universal SYBR green Supermix (Bio-Rad Laboratories, Hercules, CA). C. burnetii GE were extrapolated from a standard curve prepared using recombinant CBU1206.
