# Supplementary figures and images for: Critical Role for Molecular Iron in Coxiella burnetii Replication and Viability
Source: mSphere. 2020 Jul 22;5(4):e00458-20. doi: 10.1128/mSphere.00458-20 (PMC7376505; doi:10.1128/mSphere.00458-20)

Fig. S1

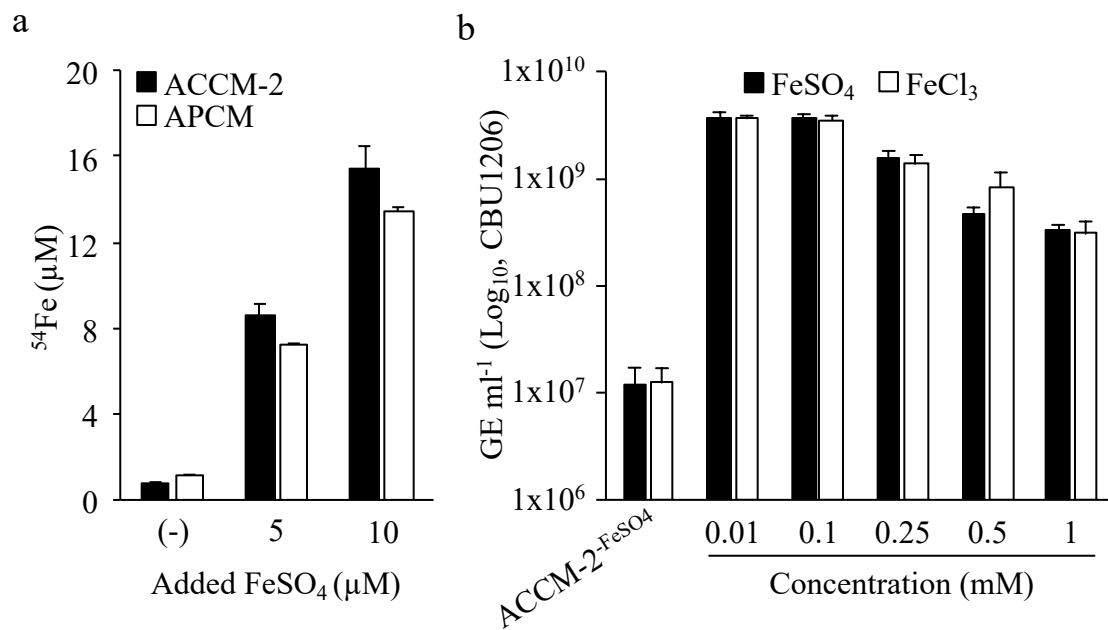

Supplement: FIG S1 [file mSphere.00458-20-sf001.pdf]

Fig. S2

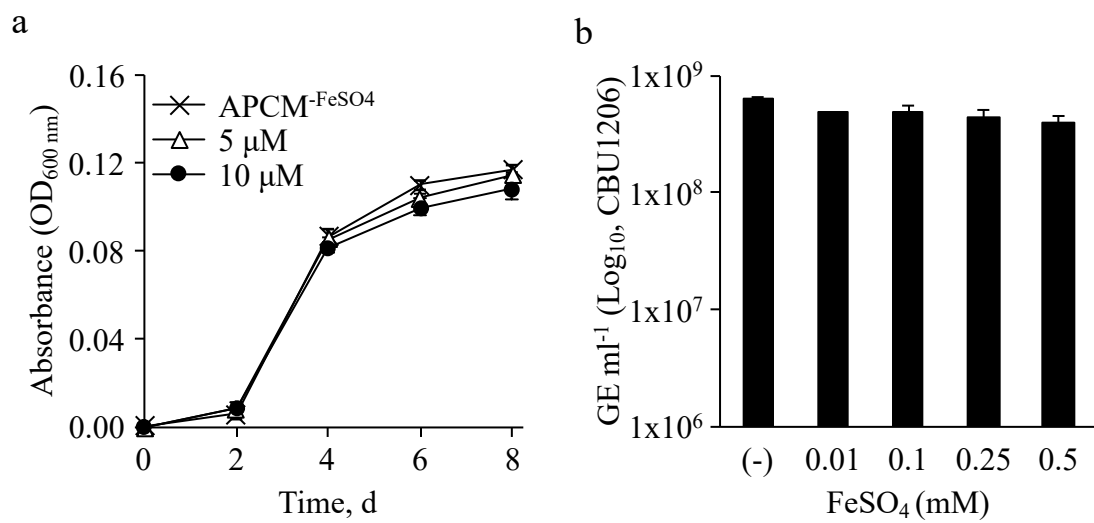

Supplement: FIG S2 [file mSphere.00458-20-sf002.pdf]

Fig. S3

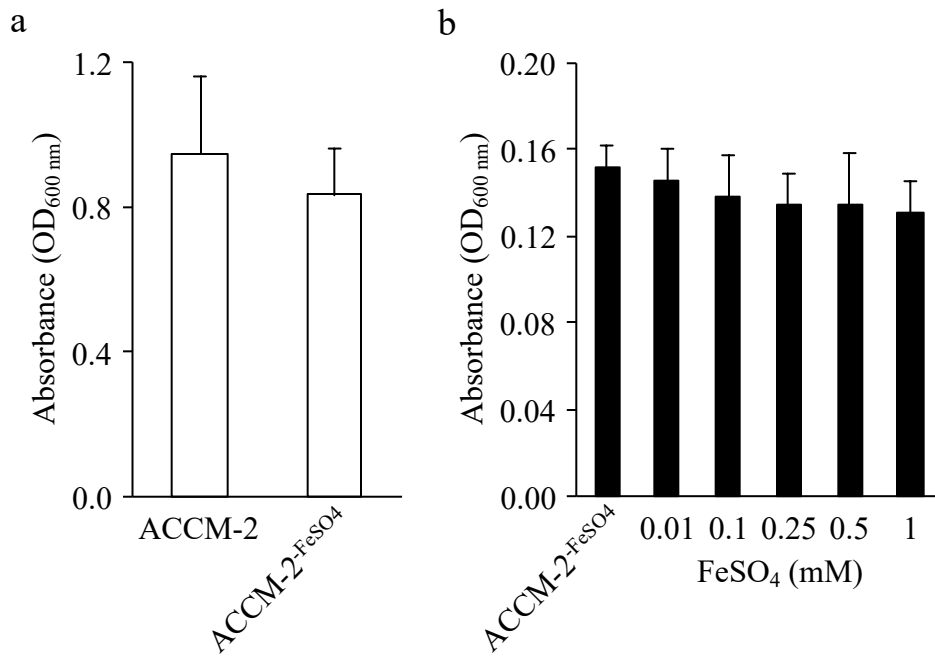

Supplement: FIG S3 [file mSphere.00458-20-sf003.pdf]

Fig. S4

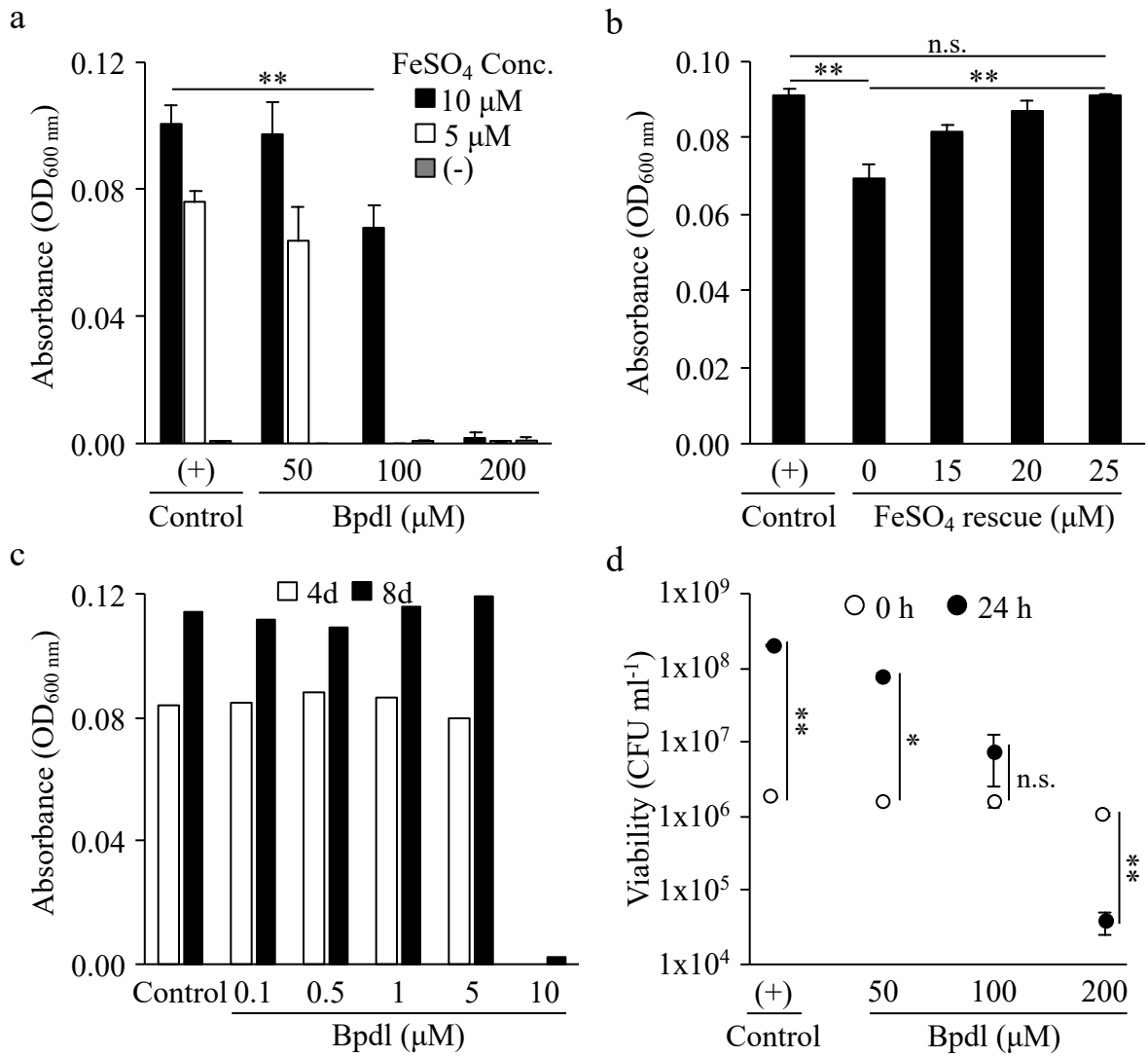

Supplement: FIG S4 [file mSphere.00458-20-sf004.pdf]

Fig. S5

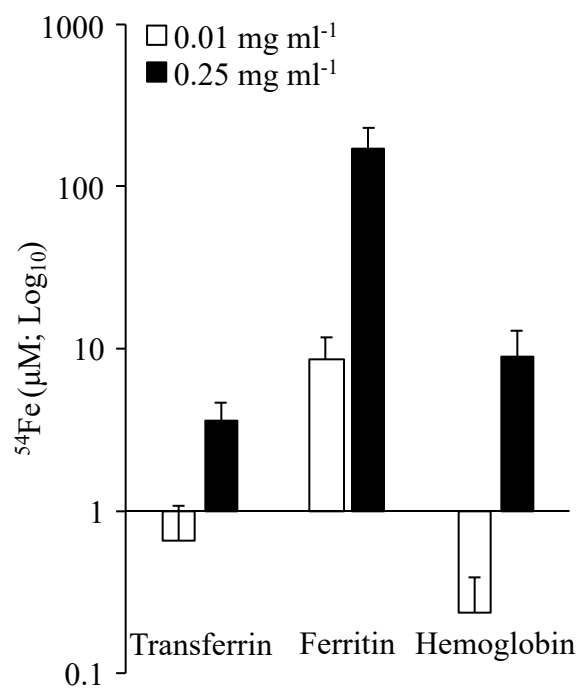

Supplement: FIG S5 [file mSphere.00458-20-sf005.pdf]

Fig. S6

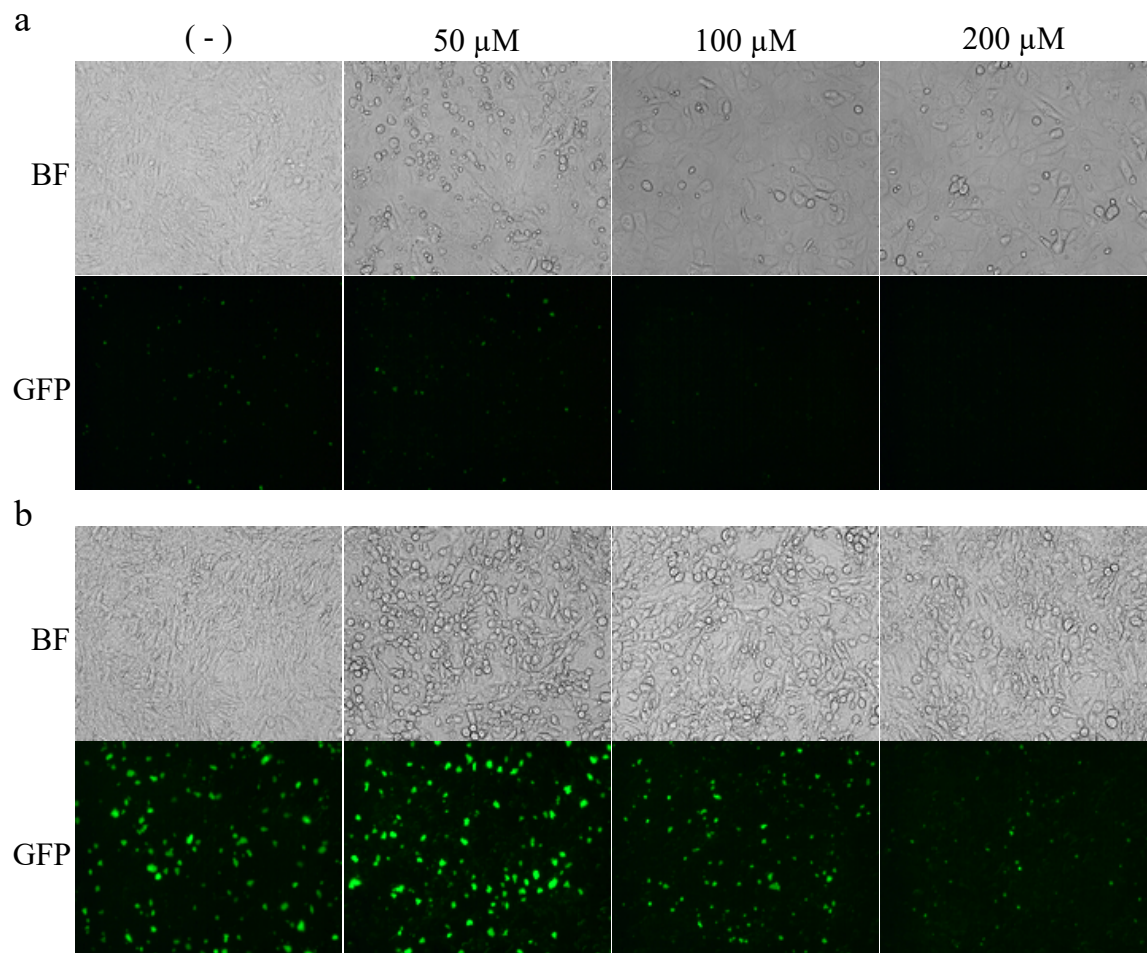

Supplement: FIG S6 [file mSphere.00458-20-sf006.pdf]

Fig. S7

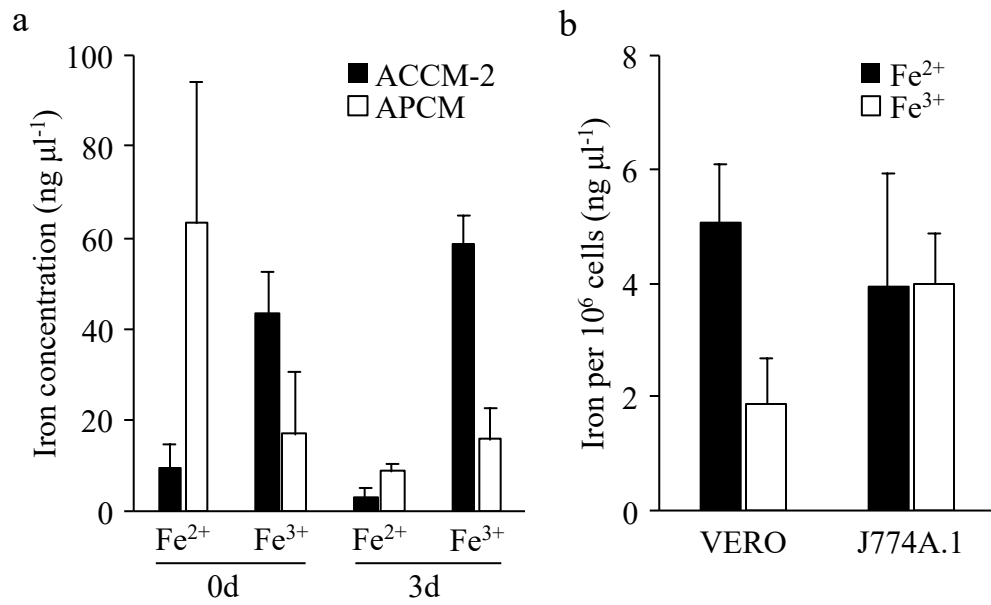

Supplement: FIG S7 [file mSphere.00458-20-sf007.pdf]
